# Patient-Reported Outcomes of Pain, Stiffness, and Fatigue Reduction in Rheumatoid and Psoriatic Arthritis With Cannabinoid Use

**DOI:** 10.7759/cureus.72366

**Published:** 2024-10-25

**Authors:** Richa Purohit, Reanne Mathai, Kathlyn Camargo Macias, Sweta Chalise, Tara Jehu, Neha Bhaskar, Neha Bhanusali

**Affiliations:** 1 Department of Internal Medicine, Concentra Urgent Care, Orlando, USA; 2 Rheumatology, University of Central Florida College of Medicine, Orlando, USA; 3 Biostatistics, University of Central Florida College of Medicine, Orlando, USA; 4 Internal Medicine, University of Alabama at Birmingham School of Medicine, Orlando, USA; 5 Internal Medicine, University of Tennessee Health Science Center, Orlando, USA

**Keywords:** cannabinoids, health surveys, outcomes, pain management, psoriatic arthritis, rheumatoid arthritis

## Abstract

Rheumatoid arthritis (RA) and psoriatic arthritis (PsA) are autoimmune conditions that can progressively destroy joints, causing chronic, often debilitating pain, and drastically affecting the quality of life. Novel pharmaceutical remedies have recently been developed, which allow for better symptom management. However, the complex pain experienced is challenging to control fully, leading this patient population to seek alternative treatments. Though cannabis has been legalized for medical use in most states in the United States, the safety and efficacy of its use in inflammatory arthritis have still not been satisfactorily established. We conducted a cross-sectional study on patients with RA and PsA who visited a rheumatology outpatient clinic from October 2019 to March 2020. We conducted a brief, voluntary, and anonymous Qualtrics survey of specific questions regarding their use of cannabinoids and their forms, sources, methods, side effects, and perceived efficacy. The survey initially involved 302 eligible candidates, but only 290 patients completed it. Among them, 84.9% (n, 247) reported a diagnosis of RA, while 15.1% (n, 44) reported PsA. Demographically, 82.3% (n, 238) were female, and 17.7% (n, 52) were male, with mean ages of 57.1 years for RA and 56.2 years for PsA. Around 16.95% (n=40) of RA and 11.63% (n=5) of PsA patients reported cannabinoid use, primarily inhaled for RA and topical/liquid for PsA. Post-cannabis use, a significant decrease in pain scale was noted (mean difference: 2.267, p < 0.001), with improvements in stiffness, fatigue, and swelling reported. Side effects were minimal, and most patients were willing to discuss cannabinoid treatment with their physician (80.9% RA [n=199], 86.4% PsA [n=38]). In conclusion, our study indicates that a notable portion of the patients with inflammatory arthritis including RA and PsA reported a history of use or ongoing cannabinoid use. Furthermore, the patients reported a short-term reduction of pain, fatigue, and swelling, though it is unclear if these findings are related to a placebo effect.

## Introduction

Rheumatoid arthritis (RA) and psoriatic arthritis (PsA) are autoimmune conditions affecting a significant number of American adults. RA affects around 1.3 million individuals, while PsA impacts approximately 500,000 individuals [1]. Both conditions involve immune-mediated inflammatory attacks on the synovial joints (with PsA also affecting tendons/ligaments along with skin), leading to chronic inflammation, pain, swelling, and stiffness, often accompanied by systemic involvement [2]. As these conditions have complex etiologies, management primarily focuses on preventing deformities, preserving joint function, and providing symptom relief. Besides the disease-modifying agents including biological agents, the standard approaches for pain control typically include topical and systemic corticosteroids, non-steroidal anti-inflammatory drugs (NSAIDs), and other analgesics [3].

Despite appropriate immunosuppressive treatment, many patients with rheumatic conditions continue to experience chronic pain, which significantly impacts their quality of life [4]. The etiology of pain in these conditions is complex and multifaceted, involving both inflammatory and non-inflammatory pathways, along with altered central regulatory mechanisms [5].

To address their pain, patients often explore various treatment modalities, including cannabinoids derived from the Cannabis plant. As a result, we hypothesize a prevalence of cannabis use among adult rheumatology patients. This study aims to contribute to the scientific discussion by investigating patient-reported outcomes concerning the effectiveness and potential of cannabinoids in addressing short- and long-term pain in the adult rheumatology population.

## Materials and methods

For this cross-sectional observational study, an anonymous, voluntary Qualtrics survey was administered to patients during their office visits between October 2019 and March 2020. The sample size calculation was based on the number of individuals who participated in the survey during the specified timeframe of the project. Efforts were made to maximize participation among individuals meeting the study's inclusion criteria during routine office visits. Nurses offered surveys on secured iPads in private examination rooms to these patients who provided written informed consent. The survey was made voluntary, and there was no further action for non-responses. Patients were given as much time as they needed to complete the survey. On average, the survey took approximately 5 minutes to complete all questions, if applicable. Institutional Review Board (IRB) approval was obtained for this study before data collection.

Inclusion criteria were individuals aged 18 or older diagnosed with RA or PsA, consenting to participate, and completing the English-language questionnaire. RA diagnosis followed the 2010 American College of Rheumatology (ACR)/European League Against Rheumatism (EULAR) classification criteria for RA, while PsA diagnosis adhered to the Classification Criteria for Psoriatic Arthritis (CASPAR) criteria, integrating clinical symptoms, physical examinations, laboratory tests, and imaging studies. Exclusion criteria included individuals who did not have a diagnosis of RA or PsA and those who faced challenges in completing the survey in English. Specifically, participants without a confirmed diagnosis of RA or PsA were excluded to ensure a focused study population with well-defined rheumatic conditions. Additionally, excluding individuals unable to complete the survey in English aimed to maintain consistency in responses and data interpretation, fostering clear and reliable findings.

The survey comprised 21 multiple-choice questions. Some questions offered multiple-choice options, while others were binary (yes/no), and some required participants to provide free-text responses. The questions aimed to gather details of patients' demographics, including age and gender. Ethnicity details were not included in the questionnaire. Further questions inquired about details of cannabis use such as form, source, method, dosage, quantified patient-reported outcomes, and adverse effects. "Current cannabis use" was determined by a positive response to the question "Do you take cannabinoids to treat your arthritis now?" Patients were asked about the duration for which they had been using cannabinoid compounds, with responses ranging from days, weeks, months, to years. They were also asked about the source they procured their cannabinoids from, with options including online, medical provider, local store, someone I know, and dispensary. Patients were allowed to choose as many sources as they wanted.

Patients were asked to select the dosage they were using, with choices including 0-200 mg/day, 200-500 mg/day, 500-1000 mg/day, over 1,000 mg/day, and "I don’t know." Pain levels were assessed using a 10-point pain scale, with 1 being equivalent to no pain and 10 being equivalent to the worst pain possible. Patients reported their pain scale before beginning the cannabinoids, after taking the cannabinoids, and over the long term after taking the cannabinoids.

Besides pain, questions also aimed to gather details about the benefits of other arthritis symptoms that patients may have derived from cannabinoid use, including stiffness, swelling, pain, fatigue, and none. Patients could choose as many options as they liked. Patients were asked if they experienced any side effects after using cannabinoids, with choices for yes/no. They were further asked if these side effects prompted them to discontinue cannabinoid use. A specific question regarding concerns with cannabinoid use included choices such as safety, side effects, hassle, addiction, and cost. Patients were allowed to choose as many choices as they liked.

The survey was designed with statistical support to optimize data quantification for rigorous analysis, including a Shapiro-Wilk test to confirm data normality. Statistical analysis, conducted using Statistical Package for the Social Sciences (SPSS) Version 28.0 (IBM Corp., Armonk, NY), included paired T-tests to compare the mean changes in reported pain scale for RA and PsA patients before and after using cannabinoid compounds, with a significance threshold set at p < 0.05. Descriptive and correlational analyses were also performed. Data collection and analysis adhered to established survey research methodology principles and followed the Strengthening the Reporting of Observational Studies in Epidemiology (STROBE) guidelines to ensure transparency and accuracy in reporting.

## Results

**Figure 1:**
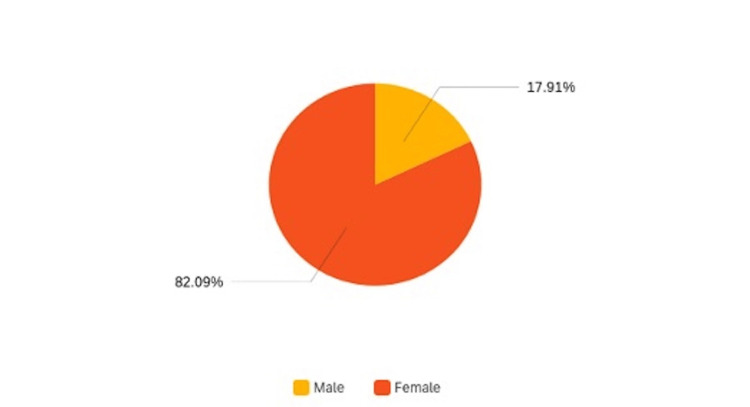
Pie chart illustrating the gender distribution of the survey population.

The survey initially involved 302 eligible candidates, but, ultimately, only 290 patients completed it. Among them, 84.9% (n=247) reported a diagnosis of RA, while 15.1% (n=44) reported PsA. Demographically, 82.3% (n=238) were female, and 17.7% (n=52) were male (Figure 1), with mean ages of 57.1 years for RA and 56.2 years for PsA. The baseline pain level for RA patients had a mean of 6.4/10 (SD 2.2), while patients with PsA reported a baseline pain level of 5.4/10 (SD 1.7).

**Figure 2:**
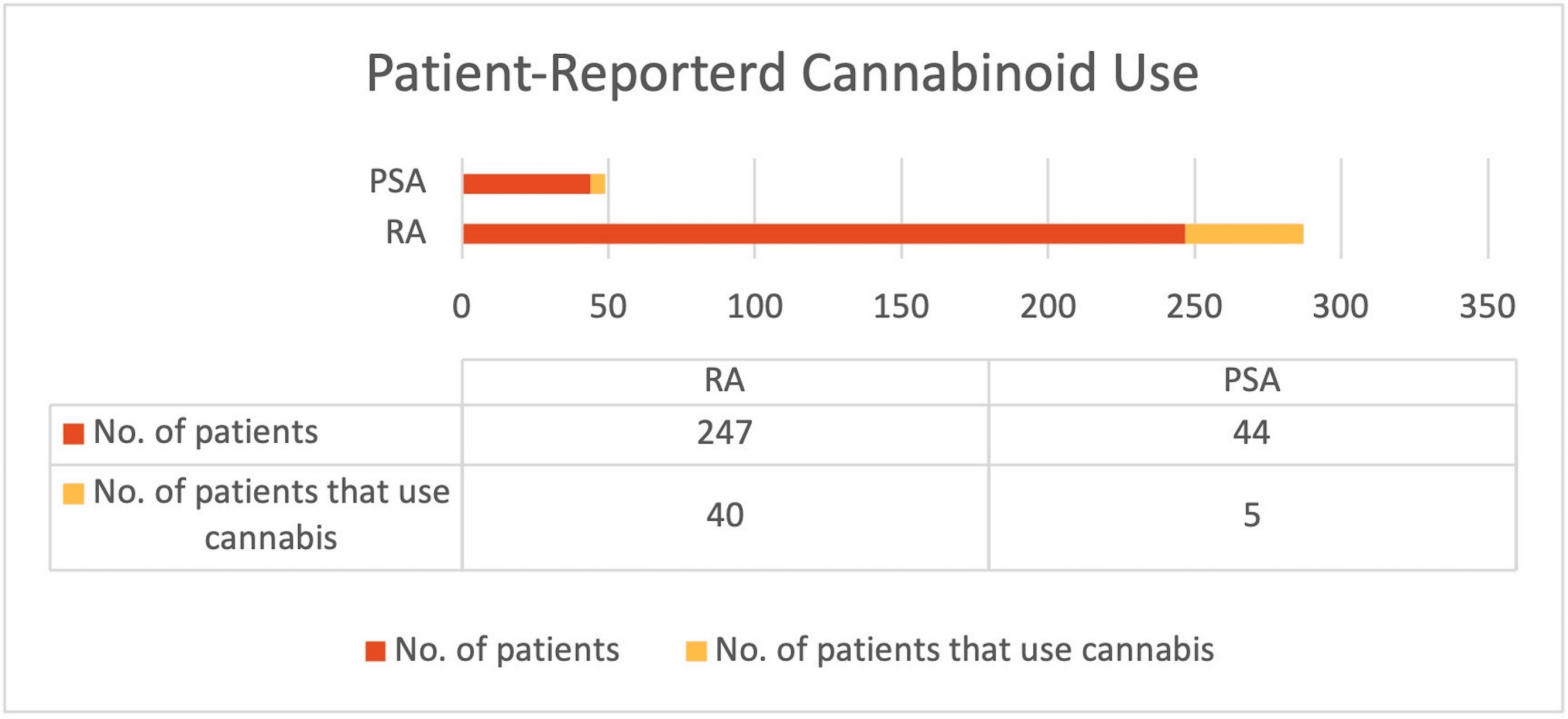
Comparative double bar graph illustrating the prevalence of cannabinoid use among our rheumatoid arthritis and psoriatic arthritis patient populations surveyed.

**Figure 3:**
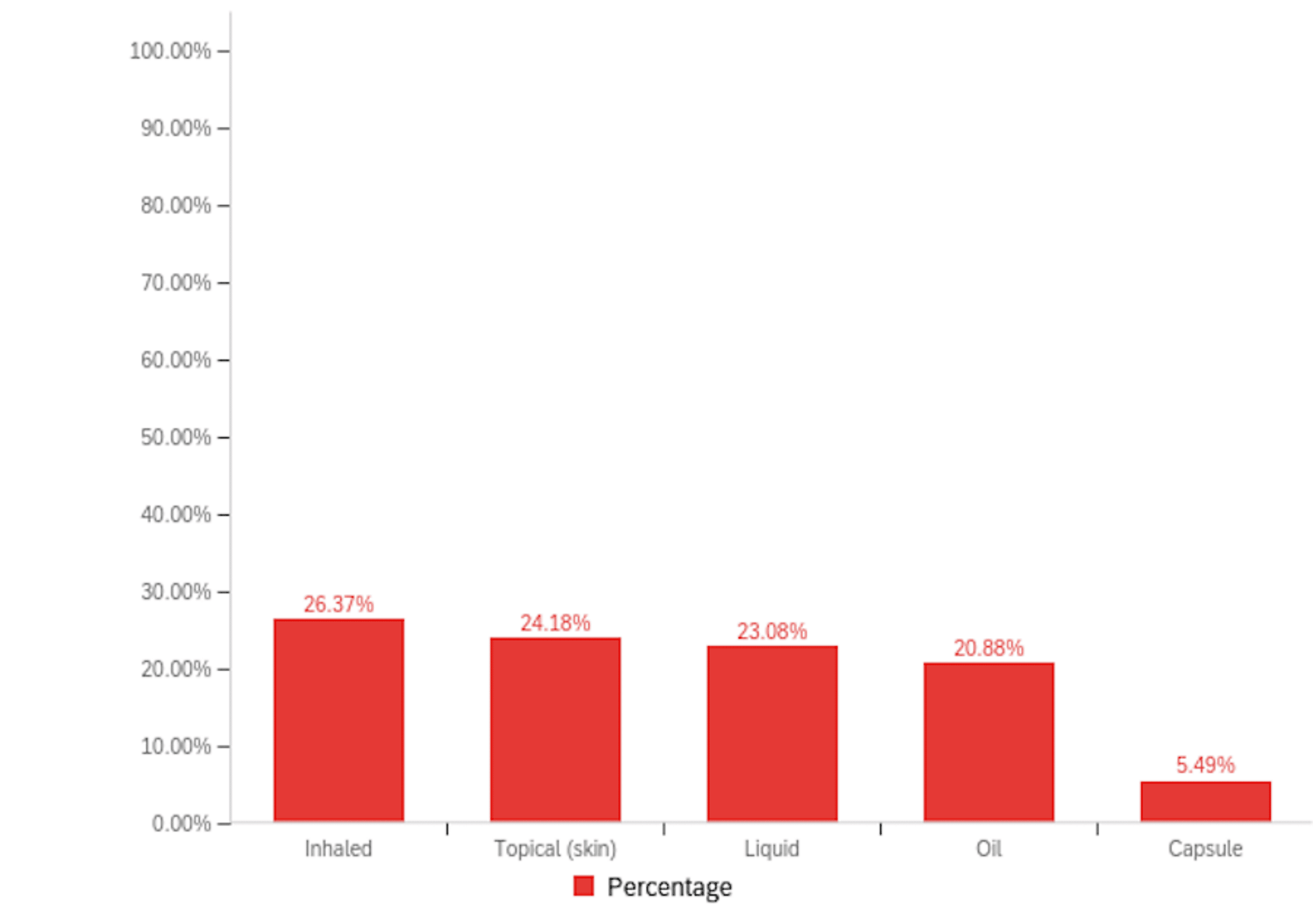
Bar graph illustrating the different forms in which the cannabinoids were used by the survey population.

**Figure 4:**
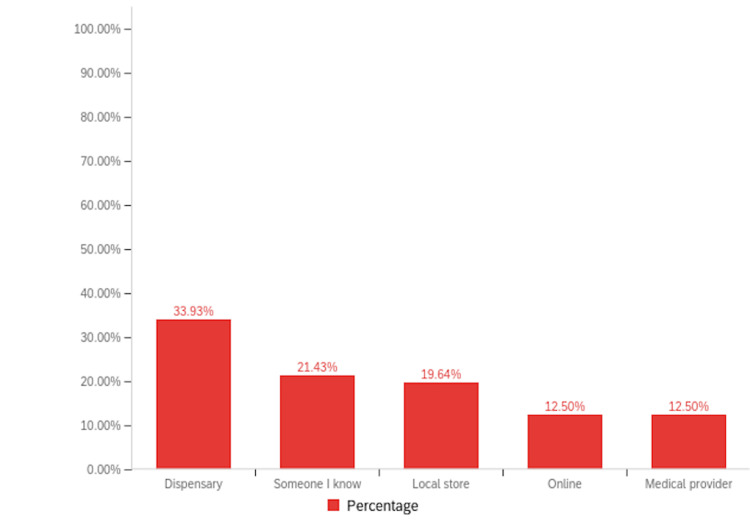
Bar graph illustrating various sources from which the survey population obtained cannabinoids.

**Figure 5:**
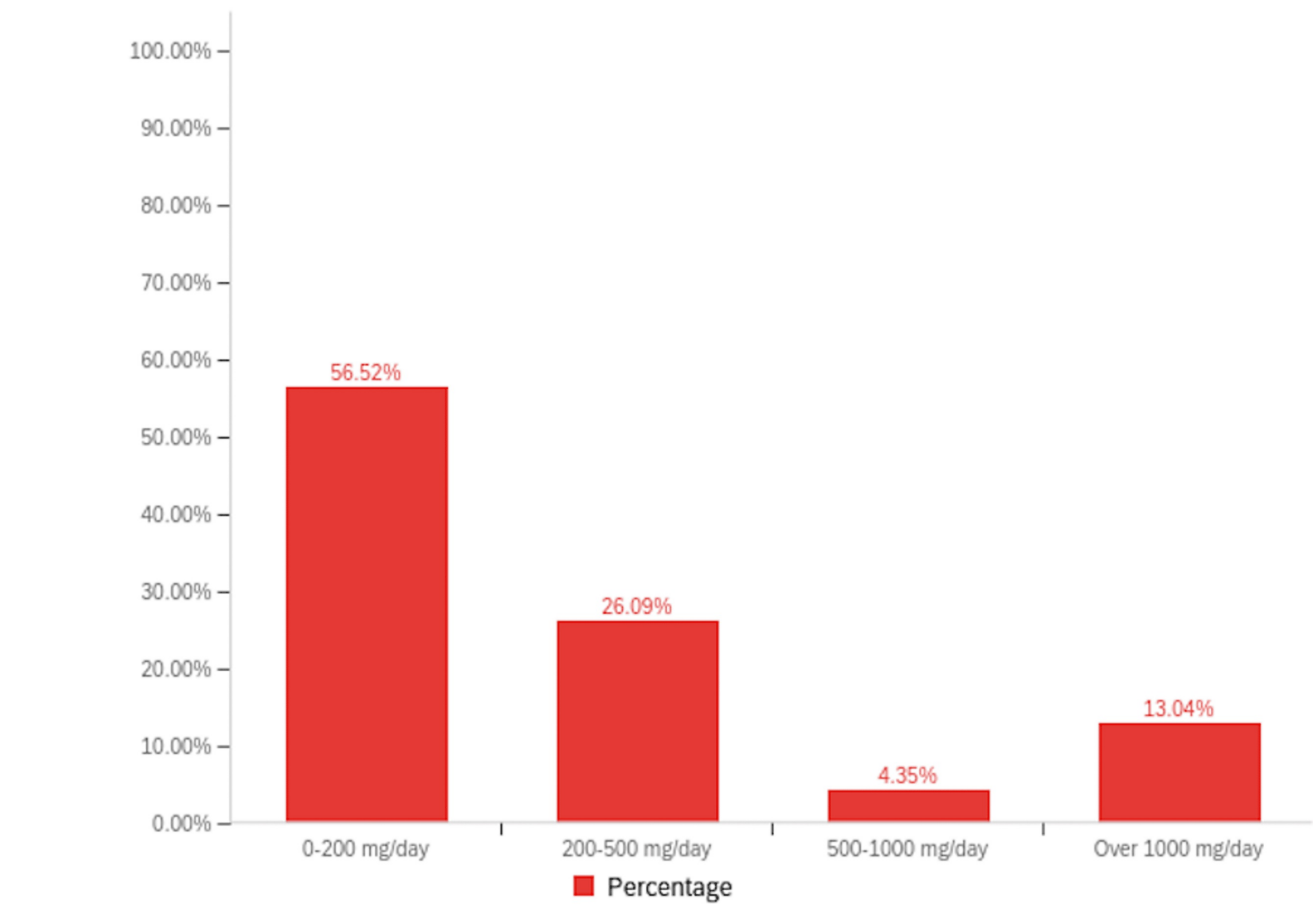
Bar graph depicting the prevalence of various dosages of cannabinoids used by the survey population.

**Figure 6:**
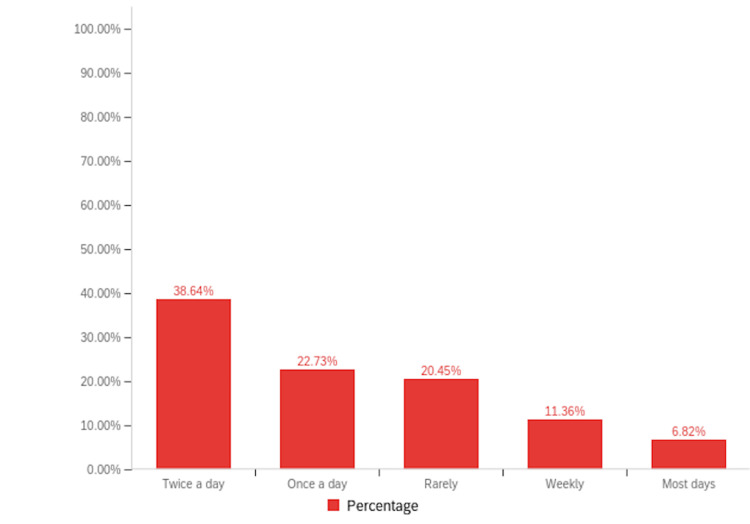
Bar graph illustrating the frequency in which the cannabinoid compounds were used by our study population.

Among patients with RA, 16.95% (n=40) reported a history of cannabinoid use to manage their symptoms, compared to 11.63% (n=5) of patients with PsA (Figure 2). Notably, inhaled cannabis was the most common form of cannabinoid used (26.37%, n=24), followed by topical formulations (24.18%, n=22) (Figure 3). When asked about the sources they procured their cannabinoid compounds from, a dispensary was the most chosen option (33.93%, n=19), followed by someone I know (21.43%, n=12) (Figure 4). Patients selected the dosages in which they were using cannabinoid compounds, with the majority choosing 0-200 mg/day (56.52%, n=13) (Figure 5). Patients were also asked about how frequently they used cannabinoids, with the majority choosing twice a day (38.64%, n=17) (Figure 6).

**Figure 7:**
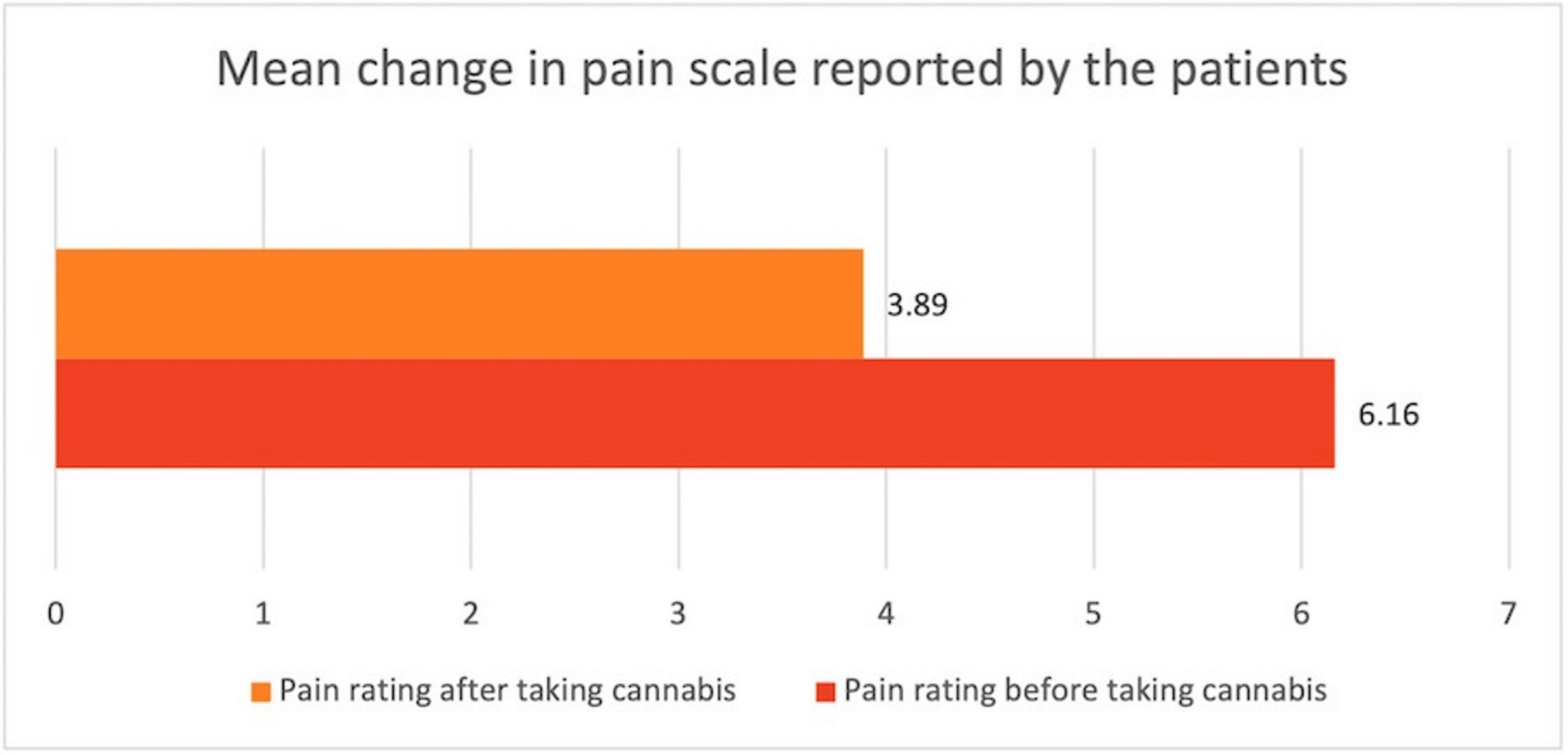
Bar graph illustrating change in the pain scale in the patients before and after the consumption of cannabinoids.

**Figure 8:**
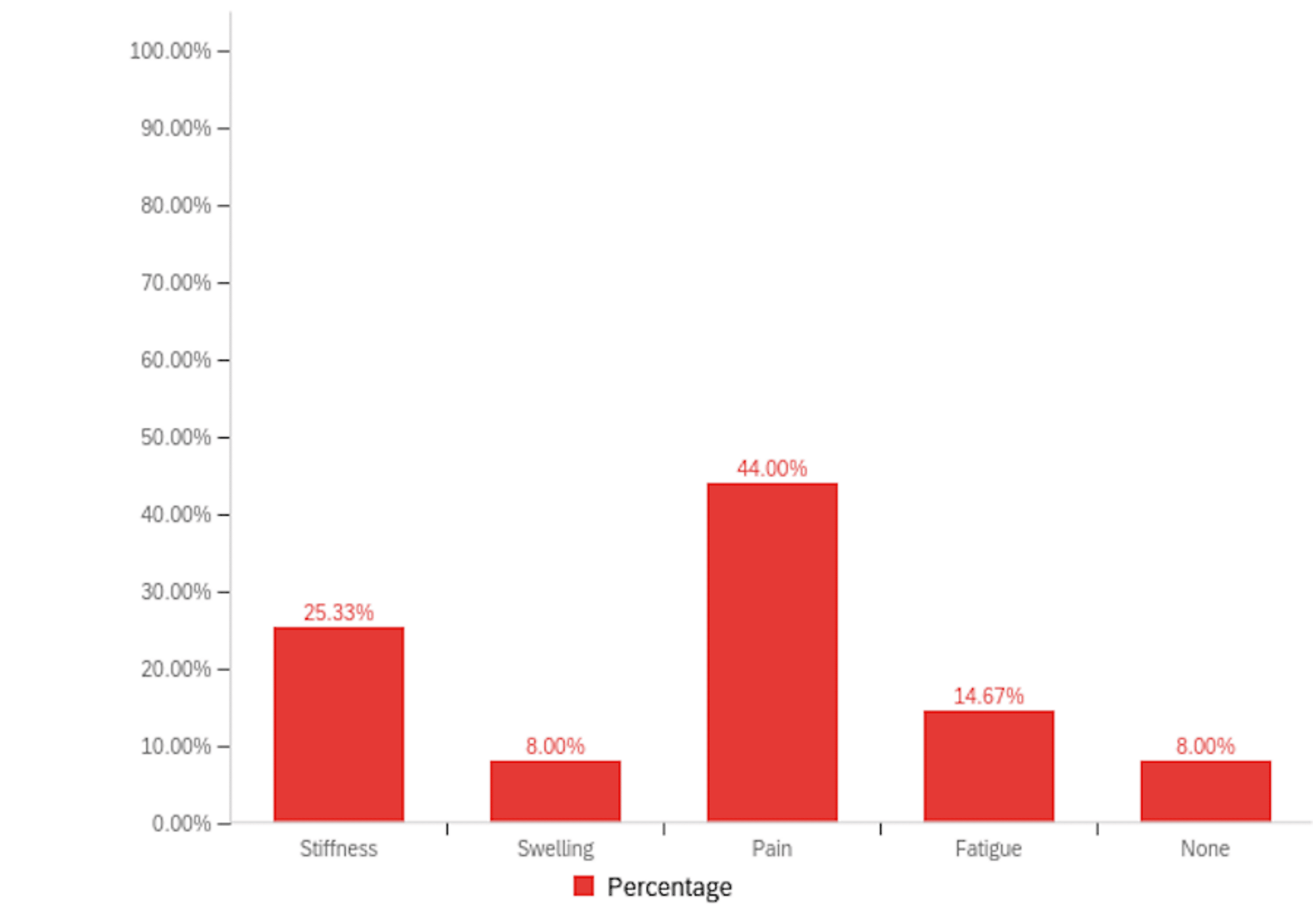
Bar graph illustrating the percentage relief in other symptoms alongside pain in the survey population.

The paired t-test was employed to assess the mean change in the pain scale before and after the intervention. Following the administration of cannabis products, there appeared to be a statistically significant decrease in patient-reported pain scale, with a mean pain scale difference of 2.267 (t = 7.717, df = 44, p < 0.001). This indicates a substantial reduction in pain severity post-taking cannabis products compared to baseline levels (Tables 1, 2, Figure 7). The patients were also encouraged to document any further symptom relief they had experienced while using cannabinoids. They were asked to choose as many options as possible from various options including pain, swelling, stiffness, fatigue, and none. Although the maximum number of participants chose pain as their first option (44%, n=32), the second most chosen option was stiffness (25.33%, n=18), followed by fatigue and swelling (Figure 8). However, it is important to note that some patients left this question blank, making it difficult to accurately interpret the numbers for this aspect.

**Table 1:** Mean change in pain scale among patients before and after cannabinoid use. This table displays the results of a paired t-test analysis examining the mean change in pain scale before and after the intervention with cannabis products. The data have been represented as N, mean, SD, and SEM. Before taking cannabis, the mean pain rating was 6.16 (SD = 2.174), based on data from 45 patients. After taking cannabis, the mean pain rating decreased to 3.89 (SD = 2.014), also based on data from 45 patients. The paired t-test yielded a statistically significant mean pain scale difference of 2.267 (t = 7.717, df = 44, p < 0.001), indicating a highly significant reduction in pain severity following the administration of cannabis products compared to baseline levels. SD, standard deviation; SEM, standard error mean

Pain	Mean	N	SD	SEM
Pain rating before taking cannabis	6.16	45	2.174	0.324
Pain rating after taking cannabis	3.89	45	2.014	0.3

**Table 2:** Results of the paired t-test analysis examining the mean change in pain scale before and after the intervention with cannabinoid products. This table displays the results of a paired t-test analysis examining the mean change in pain scale before and after the intervention with cannabis products. The data are represented as mean, SD, and SEM. A statistically significant change was observed in the patient-reported pain scale, with a mean pain scale difference of 2.267. The calculated t-value is 7.717, with 44 degrees of freedom (df), and a p-value of less than 0.001, indicating a highly significant difference. These findings suggest a substantial reduction in pain severity following the administration of cannabis products compared to baseline levels. SD, standard deviation; SEM, standard error mean

How would you rate your pain before starting to use cannabis products? How would you rate your pain right after using cannabis products?	Mean difference	Standard deviation	Standard error mean	95% confidence interval of the difference		t	df	Significance	
				Lower	Upper			One-sided p	Two-sided p
	2.267	1.97	0.294	1.675	2.859	7.717	44	<0.001	<0.001

**Figure 9:**
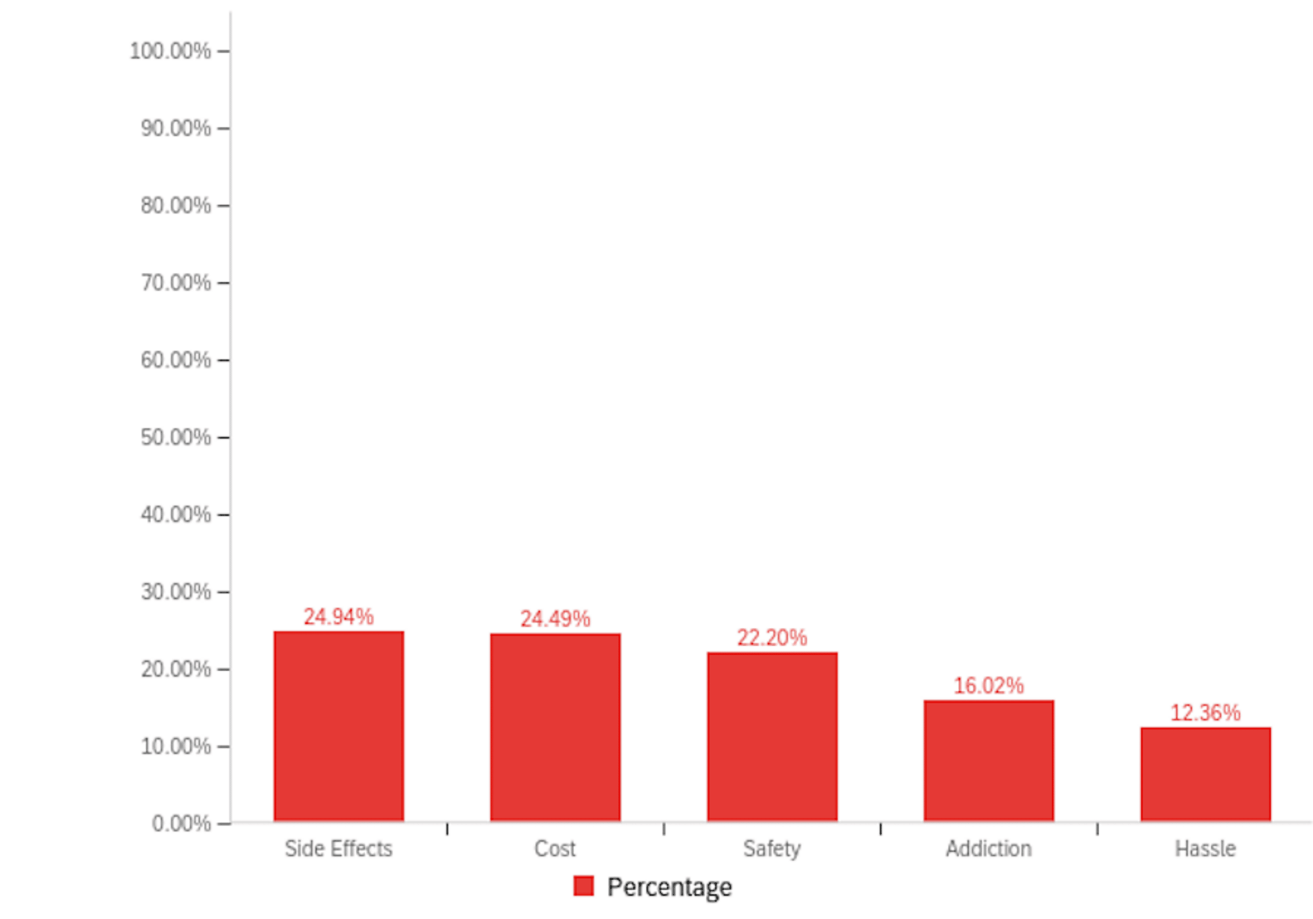
Bar graph illustrating the concerns of survey participants regarding the use of cannabinoids.

When asked to indicate whether they experienced any side effects with a yes or no question, 15.6% of RA (n=7) patients using cannabinoid preparations reported side effects; however, none of these side effects resulted in treatment discontinuation. Since they were not asked to elaborate on the type of side effects, those details are not available. In contrast, none of the patients with PsA reported experiencing side effects from cannabinoid use. All the participants in the survey (n=290) were prompted to share some concerns they had about using cannabinoids by choosing multiple options that applied, and the maximum participants chose side effects (24.94%, n=109), followed closely by cost (24.49%, n=107), and safety (22.20%, n=97), and lastly addiction (16.02%, n=70) and hassle (12.36%, n=54) (Figure 9). Interestingly, the majority of patients in both cohorts expressed a willingness to discuss cannabinoid treatment with their physician (80.9% RA [n=199], 86.4% PsA [n=38]).

## Discussion

The findings of our survey underscore the prevalence of cannabinoid use among patients with RA and PsA, with a notable proportion reporting both historical and current use. Among respondents reporting positive outcomes, pain relief was the most significant perceived benefit, followed by perceived improvements in common symptoms like stiffness, swelling, and fatigue.

The higher prevalence and persistence of cannabinoid use in the RA group, compared to the PsA group, may be attributed to the more severe baseline pain reported by RA patients. Our study also found that inhaled cannabinoids were the most common form used by RA patients, likely due to their rapid systemic absorption and quicker pain relief. In contrast, PsA patients predominantly used topical formulations, which may provide localized effects beneficial for cutaneous symptoms. These differences in the method of use suggest varying pharmacokinetics, requiring further investigation into how absorption impacts efficacy in these populations.

Despite some RA patients experiencing side effects, none discontinued cannabinoid treatment, suggesting that the perceived benefits outweighed any adverse effects. While this study did not specifically investigate dosage differences or specific side effects between these groups, variability in dosage or disease severity could explain the discrepancy. RA patients often report more severe baseline pain, potentially leading to more frequent or higher cannabinoid use, which may increase side effect frequency. Additionally, the different pathophysiological mechanisms of RA and PsA may also influence the side effect profile. Further studies would be needed to explore this relationship in more detail. However, specific side effects were not detailed in our study, although literature suggests short-term effects such as mood changes and increased appetite, and potential long-term risks such as addiction and respiratory issues [6,7].

Cannabinoids, particularly Δ9-tetrahydrocannabinol (THC) and cannabidiol (CBD), are known for their psychoactive and therapeutic effects, respectively [8]. While perceived as effective analgesics, studies have highlighted a significant placebo effect associated with their use in chronic pain management [9,10]. Moreover, cannabinoids may interact unfavorably with prescribed medications and pose adverse effects [9,10,11]. Recognizing the lack of conclusive evidence on efficacy and the potential risks of long-term use, the International Association for the Study of Pain advises caution against their use in chronic pain treatment [11]. Concerns about adverse effects and safety were the primary deterrents among respondents who had not used cannabinoids, followed by cost and perceived addiction potential. However, most respondents using cannabinoids were willing to discuss their use with a physician, emphasizing the importance of physicians being aware of patients' cannabinoid use and counseling them accordingly.

However, our study has certain limitations, notably the absence of a control group to assess the effects of cannabinoids on short and long-term pain levels. The influence of the aforementioned placebo effects raises uncertainty about how cannabinoids specifically impact chronic pain compared to alternative treatments or the natural progression of the disease. Additionally, the dataset's validity hinges on patients self-reporting subjective pain experiences, introducing the potential for confirmation bias, as respondents retrospectively reported the perceived effectiveness of a self-administered treatment. Voluntary participation may introduce selection bias, and there is a notable risk of recall bias as patients might not accurately remember pain levels before and after cannabinoid use. Although efforts were made to ensure participant anonymity, concerns about confidentiality may have led some individuals to abstain from participating. Furthermore, the survey being conducted exclusively in English could have limited the diversity of participants.

Despite these considerations, the current study emphasizes that a considerable number of RA and PsA patients are self-administering cannabinoids in various forms. It provides subjective assessments of changes in arthritic symptoms with cannabis supplementation, indicating substantial improvements in both short- and long-term pain and other secondary symptoms, with some patients even reporting complete pain resolution. Moreover, the majority of respondents expressed a willingness to discuss current or potential cannabinoid use with their physicians. This underscores the importance of initiating a dialogue between physicians and patients to enhance transparency, physician knowledge, education, and the physician-patient relationship, ultimately leading to improved patient safety and outcomes. The data supporting the findings of this study are given in Appendix A and can also be accessed through the institution’s data repository [12].

## Conclusions

In summary, our study sheds light on the self-utilization and the reported effectiveness of cannabinoids in managing symptoms associated with RA and PsA. Our data indicate that the reduction in pain was statistically significant, suggesting cannabinoids may help alleviate the pain associated with these conditions. Interestingly, the majority of patients in both groups expressed a willingness to discuss cannabinoid treatment with their physician, highlighting the importance of patient-provider communication. The data supporting the findings of this study are available upon request and can also be accessed through the institution’s data repository.

Moving forward, further investigations are needed to deepen our understanding of cannabinoid use in chronic pain management, including the incorporation of control groups and the exploration of cannabinoid use in diverse populations. More detailed examinations could elucidate specific components of pain alleviated by cannabinoids. Overall, our findings contribute to the growing literature on cannabinoid therapy for arthritis symptoms, emphasizing the need for continued research to optimize treatment strategies for affected individuals.

## Appendices

Appendix A 

**Table 3:** Survey data on patient-reported outcomes of pain, stiffness, and fatigue reduction in rheumatoid and psoriatic arthritis with cannabinoid use

Q1	Q2	Q3	Q4	Q5	Q6	Q7	Q8	Q9	Q10	Q11	Q12	Q13	Q14	Q14	Q16	Q17	Q18	Q19	Q20	Q21
Do you have Rheumatoid arthritis or Psoriatic arthritis?	Birth Year?	Gender	Have you ever used Cannabis products to treat your arthritis?	What form of Cannabis have you used to treat your condition? (select all that apply)	How long have you used Cannabis products for your arthritis?	Do you still use Cannabis products?	Where do you get your Cannabis products? (select all that apply)	How much do you take?	How often do you take it?	How would you rate your pain before starting to use Cannabis products?	How would you rate your pain right after using Cannabis products?	How would you rate your pain over the long-term with Cannabis?	Overall, how did your symptoms change with Cannabis product use?	Which symptoms, if any, were better? (select all that apply)	Which symptoms, if any, were worse? (select all that apply)	Did you have any side effects after starting to use Cannabis products?	Did these side effects stop you from using Cannabis products?	How would you currently rate your pain?	What are your concerns with Cannabis products? (select all that apply)	Are you comfortable talking about Cannabis with your doctor?
Rheumatoid arthritis	1962	Male	No															6	Safety, cost, side effects, addiction	Yes
Rheumatoid arthritis	1979	Female	No															8	Cost, side effects	Yes
Rheumatoid arthritis	1971	Female	No															2	Safety, side effects, addiction	Yes
Rheumatoid arthritis	1947	Female	Yes	Liquid	Months	Yes	Dispensary	0-200 mg/day	Twice a day	3	2	2	1: a little better	Pain	None	No		2	Cost	Yes
Rheumatoid arthritis	1939	Female	No															5	Cost	Yes
Psoriatic arthritis	1959	Male	No																	
Psoriatic arthritis	1973	Male	No															2	Safety, cost, side effects, addiction	Yes
Psoriatic arthritis	1956	Male	No															2	Safety, hassle, side effects, addiction	Yes
Rheumatoid arthritis	1963	Male	No															2	Safety, side effects, addiction	Yes
Rheumatoid arthritis	1962	Male	No															2		Yes
Rheumatoid arthritis	1947	Female	No															5	Addiction	Yes
Rheumatoid arthritis	1977	Female	No															8		No
Rheumatoid arthritis	1946	Female	Yes	Topical (skin), liquid	Weeks	No	Someone I know		Most days	9	9	9	0: no change	None	None	No		1 = No Pain		Yes
Rheumatoid arthritis	1990	Female	No															9	Side effects	Yes
Rheumatoid arthritis	1952	Female	No															7	Side effects, addiction	Yes
Rheumatoid arthritis	1972	Female	Yes	Topical (skin), oil	Days	No	Local store		Rarely	3	1 = No Pain	2	1: a little better	Pain	None	No		1 = No Pain	Cost	Yes
Rheumatoid arthritis	1983	Male	No															4	Cost	Yes
Rheumatoid arthritis	1961	Female	No															8	Cost	Yes
Rheumatoid arthritis	1963	Female	Yes	Liquid	Weeks	No	Local store	0-200 mg/day	Twice a day	8	7	7	0: no change	None	None	No		3	Cost	Yes
Psoriatic arthritis	1965	Female	No															5	Addiction	Yes
Psoriatic arthritis	1992	Female	No															7	Safety, side effects, addiction	No
Rheumatoid arthritis	1952	Male	Yes	Oil	Weeks	Yes	Online		Twice a day											
Rheumatoid arthritis	1965	Female	No															4	Cost	Yes
Rheumatoid arthritis	1962	Female	Yes	Topical (skin), oil, inhaled	Years	Yes	Online, Dispensary		Twice a day	9	7	7	1: a little better	Stiffness	None	No		10 = Worst Pain Possible	Cost	Yes
Rheumatoid arthritis	1960	Female	No															4	Cost	Yes
Rheumatoid arthritis	1936	Female	No																	
Rheumatoid arthritis	1948	Female	No															6	Side effects	Yes
	1961	Female	Yes	Topical (skin)	Weeks	Yes	Local store	200-500 mg/day	Once a day	3	1 = No Pain	2	1: a little better	Stiffness, pain	Stiffness, None	No		3		Yes
Rheumatoid arthritis	1939	Male	No															1 = No Pain	Addiction	No
Rheumatoid arthritis	1972	Female	No															2	Cost	Yes
Rheumatoid arthritis	1972	Female	No															5	Side effects	Yes
Rheumatoid arthritis	1963	Male	Yes	Topical (skin), liquid, oil, inhaled	Months	Yes	Someone I know	0-200 mg/day	Rarely	7	7	7	0: no change	None	None	Yes	No	7	Side effects	Yes
Rheumatoid arthritis	1967	Female	No															7	Cost, side effects	Yes
Rheumatoid arthritis	1956	Female	No															6	Side effects, addiction	Yes
Psoriatic arthritis	1956	Female	No															2	Hassle, side effects	Yes
Psoriatic arthritis	1997	Female	No															6	Safety, side effects	Yes
Rheumatoid arthritis	1954	Female	Yes	Topical (skin), inhaled	Months	Yes	Someone I know		Weekly	6	5	6	1: a little better	Stiffness, pain	None	No		5	Cost	Yes
Rheumatoid arthritis	1959	Female	Yes	Liquid, inhaled	Years	Yes	Dispensary, Medical provider	0-200 mg/day	Twice a day	6	2	4	1: a little better	Stiffness, pain	None	Yes	No	4	Hassle, side effects, addiction	Yes
Rheumatoid arthritis	1971	Female	No															1 = No Pain		Yes
Rheumatoid arthritis	1987	Male	No															2	Safety, side effects	Yes
Rheumatoid arthritis	1970	Female	No															1 = No Pain	Addiction	Yes
Rheumatoid arthritis	1963	Male	Yes	Liquid	Years	Yes	Online	Over 1000 mg/day	Twice a day	6	4	5	1: a little better	Pain	None	No		5	Safety, cost	Yes
Rheumatoid arthritis	1993	Female	Yes	Capsule	Weeks	No	Local store		Most days	5	5	5	0: no change	None	None	No		6	Hassle	Yes
Rheumatoid arthritis	1945	Female	No																	No
Rheumatoid arthritis	1963	Female	No															1 = No Pain	Cost	Yes
Rheumatoid arthritis	1964	Female	No															5	Hassle	No
Rheumatoid arthritis	1975	Female	Yes	Topical (skin), oil	Months	Yes	Local store, Medical provider	0-200 mg/day	Once a day	5	4	4	1: a little better	Pain, fatigue	None	Yes	No	3	Safety, cost, side effects	Yes
Rheumatoid arthritis	1982	Female	No															1 = No Pain	Cost	Yes
Rheumatoid arthritis	1960	Male	No															2	Safety, hassle, side effects	Yes
Rheumatoid arthritis	1965	Female	No																Safety, side effects, addiction	Yes
Psoriatic arthritis	1979	Male	No															3		Yes
Rheumatoid arthritis	1960	Female	No															7	Safety, hassle, cost, side effects, addiction	Yes
Rheumatoid arthritis	1952	Female	No															1 = No Pain	Safety, side effects, addiction	Yes
Rheumatoid arthritis	1942	Female	No															4		Yes
Rheumatoid arthritis	1967	Female	No															8	Safety	Yes
Psoriatic arthritis	1948	Female	No															2	Hassle	Yes
Rheumatoid arthritis	1961	Female	No															3	Safety, hassle, cost, side effects, addiction	Yes
Rheumatoid arthritis	1956	Female	No															8	Side effects	Yes
Rheumatoid arthritis	1981	Female	Yes	Liquid, oil, inhaled	Months	No	Dispensary	0-200 mg/day	Rarely	6	5	6	0: no change	Pain, fatigue	None	Yes	No	6		No
	1972	Female	No															9		Yes
Rheumatoid arthritis	1960	Female	No															2	Safety, hassle, cost, side effects, addiction	Yes
Rheumatoid arthritis	1990	Female	No															8	Safety, cost, side effects, addiction	Yes
Rheumatoid arthritis	1951	Female	No															7	Safety, hassle, cost, side effects	No
Rheumatoid arthritis	1965	Female	No															5	Side effects	Yes
Rheumatoid arthritis	1947	Female	No															7		Yes
Psoriatic arthritis	1958	Male	No															1 = No Pain	Safety, hassle, cost, side effects, addiction	Yes
Rheumatoid arthritis	1994	Female	No															2	Safety, side effects	Yes
Rheumatoid arthritis	1942	Female	No															3	Side effects	No
Rheumatoid arthritis	1967	Female	Yes	Oil, inhaled	Weeks	Yes	Dispensary, Medical provider	500-1000 mg/day	Twice a day	10 = Worst Pain Possible	6	6	1: a little better	Stiffness, swelling, pain, fatigue	None	No		6	Safety, cost, side effects	Yes
Rheumatoid arthritis	1958	Female	No															3	Side effects	Yes
Rheumatoid arthritis	1950	Female																		
	1958	Female	No															1 = No Pain	Safety	Yes
Rheumatoid arthritis	1971	Male	No															8	Hassle	Yes
Rheumatoid arthritis	1974	Female	No															5	Cost	Yes
Rheumatoid arthritis	1959	Female	No															1 = No Pain	Safety, cost, side effects, addiction	Yes
Rheumatoid arthritis	1989	Female	No															3	Safety, side effects, addiction	No
Rheumatoid arthritis	1950	Female	No															1 = No Pain		Yes
Rheumatoid arthritis	1947	Female																		
Rheumatoid arthritis	1963	Female	No															5		No
Rheumatoid arthritis	1979	Female	No															1 = No Pain	Safety, side effects, addiction	Yes
Psoriatic arthritis	1951	Male	No															6	Hassle	Yes
Psoriatic arthritis	1959	Male	No															7	Side effects	Yes
Rheumatoid arthritis	1958	Female	No															8	Safety, hassle, cost, side effects, addiction	No
Rheumatoid arthritis	1939	Female	No															7		Yes
Rheumatoid arthritis	1960	Female	Yes	Inhaled	Months	Yes	Dispensary		Rarely	8	3	3	0: no change	Pain	None	No		4	Safety	Yes
Rheumatoid arthritis	1955	Female	No															3	Safety, side effects	No
Rheumatoid arthritis	1986	Male	Yes	Inhaled	Days	Yes	Someone I know		Rarely	4	2		1: a little better	Stiffness, pain, fatigue	None	No		4	Safety, hassle, cost	Yes
Psoriatic arthritis	1976	Female	No															3		
Rheumatoid arthritis	1997	Female	No															3	Safety	No
Rheumatoid arthritis	1962	Female	No															8	Safety, cost, side effects, addiction	Yes
Rheumatoid arthritis	1959	Female	No															7		Yes
Rheumatoid arthritis	1948	Female	No															5	Safety, cost, side effects	No
Rheumatoid arthritis	1958	Female	No															2	Cost, addiction	Yes
Rheumatoid arthritis	1963	Female	No															10 = Worst Pain Possible		Yes
Rheumatoid arthritis	1947	Female	No															4	Safety, cost, side effects, addiction	Yes
Rheumatoid arthritis	1960	Female	No															2		Yes
Rheumatoid arthritis	1974	Female	No															2	Safety, addiction	No
Psoriatic arthritis	1970	Male	No															8		Yes
Rheumatoid arthritis	1943	Female	No															4		Yes
Rheumatoid arthritis	1961	Female	No															5	Safety	Yes
Rheumatoid arthritis	1949	Male	No															1 = No Pain	Safety	Yes
Rheumatoid arthritis	1962	Female	No															4	Cost	Yes
Rheumatoid arthritis	1959	Male	Yes	Inhaled	Days	No	Someone I know		Rarely	7	4		1: a little better	Pain	None	No		7		Yes
Rheumatoid arthritis	1988	Female	No															2	Safety	Yes
Rheumatoid arthritis	1958	Female	No															6	Cost	Yes
Rheumatoid arthritis	1979	Female	No															7	Safety	Yes
Rheumatoid arthritis	1945	Female	No															3	Safety, cost, side effects, addiction	Yes
Rheumatoid arthritis	1967	Female	No															1 = No Pain	Side effects	Yes
Rheumatoid arthritis	1966	Female	No															1 = No Pain	Safety, hassle, cost, side effects, addiction	Yes
Rheumatoid arthritis	1976	Female	No															6	Cost, side effects	Yes
Rheumatoid arthritis	1970	Female	No															3	Safety, side effects, addiction	Yes
Rheumatoid arthritis	1966	Female	No															1 = No Pain		No
Rheumatoid arthritis	1958	Female	No															3	Side effects	Yes
Psoriatic arthritis	1956	Female	No															7	Safety, cost, side effects	Yes
Rheumatoid arthritis	1962	Male	No															7	Cost	Yes
Rheumatoid arthritis	1967	Female	No															7	Hassle, cost, side effects	Yes
Rheumatoid arthritis	1984	Female	No															6	Safety, cost, side effects, addiction	Yes
Rheumatoid arthritis	1953	Female	No															5	Cost	Yes
Rheumatoid arthritis	1944	Female																		
Psoriatic arthritis	1962	Female	No															2	Hassle, cost	Yes
Rheumatoid arthritis	1955	Female	No															3	Cost	Yes
Rheumatoid arthritis	1974	Male	No															5		Yes
Rheumatoid arthritis	1969	Female	No															2		Yes
Rheumatoid arthritis	1962	Male	No																	
Psoriatic arthritis	1931	Female	No															4		No
Psoriatic arthritis	1946	Female	No															4	Safety	Yes
Rheumatoid arthritis	1950	Female	No															1 = No Pain		Yes
Rheumatoid arthritis	1957	Female	Yes	Topical (skin), capsule, oil, inhaled	Months	Yes	Dispensary	200-500 mg/day	Twice a day	4	2	3	1: a little better	Pain	Fatigue	No		3	Safety	No
Psoriatic arthritis	1976	Female	Yes	Liquid	Weeks	No	Local store		Twice a day	8	6	6	1: a little better	Pain	None	No		6	Safety, side effects	Yes
Rheumatoid arthritis	1974	Male	No															2		Yes
Rheumatoid arthritis	1972	Female	No															1 = No Pain	Safety, side effects, addiction	Yes
Rheumatoid arthritis	1948	Female	No															2	Addiction	No
Rheumatoid arthritis	1959	Female	Yes	Topical (skin)	Months	Yes	Online	0-200 mg/day	Once a day	5	5	5	1: a little better	Pain	Pain	No		5	Safety	Yes
Rheumatoid arthritis	1972	Female	No															3	Safety, hassle, cost, side effects, addiction	Yes
Rheumatoid arthritis	1959	Female	No																Safety, hassle, cost, side effects, addiction	Yes
Rheumatoid arthritis	1958	Female	No															6		
Rheumatoid arthritis	1956		No																Safety, hassle, cost, side effects, addiction	No
Rheumatoid arthritis	1976	Female	No															2	Side effects	Yes
Rheumatoid arthritis	1980	Female	Yes	Topical (skin), liquid, oil	Weeks	No	Dispensary	0-200 mg/day	Weekly	6	2		2: much better	Stiffness, pain	Fatigue	No		3	Cost	Yes
Rheumatoid arthritis	1965	Female	No															2	Safety	Yes
Rheumatoid arthritis	1972	Female	No															2	Side effects	Yes
Rheumatoid arthritis	1976	Female	No															3		Yes
Rheumatoid arthritis	1967	Female	Yes	Topical (skin), inhaled	Years	Yes	Dispensary, Medical provider	0-200 mg/day	Rarely	2	1 = No Pain	2	2: much better	Fatigue	None	No		2	Safety	Yes
Rheumatoid arthritis	1939	Female																		
Rheumatoid arthritis	1947	Female	No															5	Side effects, addiction	Yes
Rheumatoid arthritis	1946	Male	Yes	Oil	Weeks	No	Local store		Twice a day	3	3	3	0: no change	None	None	No		3	Hassle	Yes
Rheumatoid arthritis	1950	Male	No															2	Cost	Yes
Rheumatoid arthritis	1962	Female	No															6	Safety, side effects	Yes
Rheumatoid arthritis	1937	Female	No															8		Yes
Rheumatoid arthritis	1960	Female	No															2	Safety, hassle, cost, side effects, addiction	Yes
Rheumatoid arthritis	1955	Male	No															1 = No Pain	Addiction	Yes
Rheumatoid arthritis	1935	Female		Liquid	Days	No	Online	0-200 mg/day	Once a day	9	9		0: no change	None	None	No		3	Safety, hassle, cost	Yes
Rheumatoid arthritis	1936	Female	No															10 = Worst Pain Possible		Yes
Rheumatoid arthritis	1974	Female	No															3	Safety, hassle, cost, side effects	Yes
Rheumatoid arthritis	1939	Female	No															4	Hassle, cost, side effects	Yes
Rheumatoid arthritis	1963	Female	Yes	Topical (skin), liquid	Weeks	No	Local store		Rarely	8	5	8	1: a little better	Stiffness, pain	None	No		1 = No Pain	Safety, side effects	Yes
Rheumatoid arthritis	1963	Female	Yes	Topical (skin), inhaled	Years	Yes	Dispensary, Medical provider		Twice a day	8	4	4	2: much better	Stiffness, pain	Stiffness, pain	No		6	Cost	Yes
Rheumatoid arthritis	1968	Female	No															2	Safety, hassle, side effects, addiction	Yes
Rheumatoid arthritis	1961	Female	Yes	Topical (skin)	Months	Yes	Local store			5	5	5	0: no change	Stiffness	None	No		5		Yes
Rheumatoid arthritis	1970	Female	Yes	Inhaled	Years	Yes	Dispensary	200-500 mg/day	Twice a day	8	4	4	2: much better	Pain, fatigue	None	Yes	No	4	Cost	Yes
Rheumatoid arthritis	1964	Female	No															6	Safety, cost, addiction	Yes
Psoriatic arthritis	1960	Female	No															6	Cost, side effects, addiction	Yes
Rheumatoid arthritis	1948	Female																		
Rheumatoid arthritis	1962	Female	No															7	Cost	Yes
Rheumatoid arthritis	1947	Female	No															4		Yes
Rheumatoid arthritis	1966	Female	Yes	Liquid, inhaled	Months	Yes	Someone I know		Once a day	10 = Worst Pain Possible	6	6	1: a little better	Stiffness	Pain, fatigue	No		6		Yes
Rheumatoid arthritis	1957	Female	No															2	Safety, side effects	No
Rheumatoid arthritis	1969	Female	No															7	Side effects	Yes
Rheumatoid arthritis	1969	Female	No															2		Yes
Rheumatoid arthritis	1940	Female	No															5	Safety	Yes
Rheumatoid arthritis	1945	Female	No															7	Safety, cost, side effects	Yes
Rheumatoid arthritis	1986	Female	No															3	Safety	Yes
Rheumatoid arthritis	1967	Female	No															2	Side effects, addiction	Yes
Rheumatoid arthritis	1944	Female	No															1 = No Pain	Safety, hassle, cost, side effects, addiction	Yes
Rheumatoid arthritis	1971	Female	No															2	Cost	Yes
Rheumatoid arthritis	1954	Female	No															7	Cost	Yes
Rheumatoid arthritis	1954	Female	No															8	Cost	Yes
Rheumatoid arthritis	1964	Female	No															2	Side effects, addiction	No
Rheumatoid arthritis	1965	Female	No															7	Cost	Yes
Rheumatoid arthritis	1953	Female	No															2	Hassle, cost	Yes
Rheumatoid arthritis	1948	Female	No															2	Safety, hassle, cost, side effects, addiction	Yes
Psoriatic arthritis	1968	Male	No															3		No
Rheumatoid arthritis	1945	Female	No																	
Rheumatoid arthritis	1958	Female	No															10 = Worst Pain Possible	Hassle	Yes
Psoriatic arthritis	1954	Female	No															1 = No Pain	Safety	Yes
Rheumatoid arthritis	1981	Male	No															2	Cost	Yes
Rheumatoid arthritis	1967	Female	No															7		Yes
Rheumatoid arthritis	1969	Female																		
Psoriatic arthritis		Female																		Yes
Psoriatic arthritis	1971	Female	No																Safety, hassle, side effects, addiction	Yes
Rheumatoid arthritis	1964	Female	No															2	Safety, side effects	Yes
Rheumatoid arthritis	1951	Male	No															2		Yes
Rheumatoid arthritis	1977	Female	Yes	Topical (skin), capsule, liquid, oil, inhaled	Years	No	Online, Dispensary, Medical provider	0-200 mg/day	Twice a day	10 = Worst Pain Possible	2	6	2: much better	Stiffness, swelling, pain, fatigue	None	No		3	Cost	Yes
Psoriatic arthritis	1948	Female	No															3	Side effects	Yes
Rheumatoid arthritis	1975	Female	No															8	Side effects, addiction	Yes
Rheumatoid arthritis	1959	Male	No															1 = No Pain	Safety, side effects, addiction	Yes
Rheumatoid arthritis	1972	Female	No															2	Cost, side effects, addiction	Yes
Rheumatoid arthritis	1959	Female	No															7	Safety, side effects	Yes
Rheumatoid arthritis	1960	Female	No															5	Side effects, addiction	Yes
Rheumatoid arthritis	1994	Female	Yes	Topical (skin), liquid, inhaled	Years	Yes	Someone I know	0-200 mg/day	Weekly	6	3	3	1: a little better	Stiffness, pain, fatigue	None	No		3	Safety, hassle, cost, side effects	Yes
Rheumatoid arthritis	1955	Female	No															4	Cost, side effects	Yes
Rheumatoid arthritis	1952	Male	No															8		Yes
Rheumatoid arthritis	1959	Female	No															6	Hassle, cost, side effects	Yes
Rheumatoid arthritis	1959	Female	No															1 = No Pain	Safety, cost, side effects, addiction	Yes
Rheumatoid arthritis	1960	Female	No															5	Cost	Yes
Rheumatoid arthritis	1958	Female	No															9	Safety, cost, addiction	Yes
Rheumatoid arthritis	1948	Female																		
Rheumatoid arthritis	1964	Female																		
Rheumatoid arthritis	1986	Female	No															4	Safety, cost, side effects, addiction	Yes
Rheumatoid arthritis	1966	Female	No															1 = No Pain	Safety, side effects, addiction	No
Rheumatoid arthritis	1969	Female	Yes	Topical (skin), liquid, oil	Months	Yes	Local store	Over 1000 mg/day	Once a day	8	3	2	2: much better	Stiffness, swelling, pain	None	No		2		Yes
Psoriatic arthritis	1955	Female	No															7	Cost	Yes
Rheumatoid arthritis	1965	Female	No															6		Yes
Rheumatoid arthritis	1947	Female	No															3	Side effects	Yes
Rheumatoid arthritis	1946	Female	Yes	Liquid, oil		No				8	8	8	0: no change			No		8	Safety, cost	Yes
Rheumatoid arthritis	1963	Male	No															1 = No Pain		No
Rheumatoid arthritis	1972	Female	No															1 = No Pain		Yes
Psoriatic arthritis	1967	Female	Yes	Topical (skin), liquid, oil, inhaled	Months	Yes	Dispensary	200-500 mg/day	Twice a day	6	3	3	1: a little better	Pain, fatigue	None	No		3	Cost	Yes
Rheumatoid arthritis	1993	Female																		
Rheumatoid arthritis	1977	Male	No															2		No
Psoriatic arthritis	1966	Female	No															5	Addiction	Yes
Rheumatoid arthritis	1946	Female	No															4	Safety, cost, side effects, addiction	Yes
Rheumatoid arthritis	1945	Female	No															1 = No Pain	Safety, side effects, addiction	Yes
Psoriatic arthritis	1997	Male	No															1 = No Pain	Safety, side effects, addiction	No
Rheumatoid arthritis	1947	Male	No															2		Yes
Rheumatoid arthritis	1962	Female	No															4	Cost	Yes
Rheumatoid arthritis	1960	Female	No															4	Safety, side effects	Yes
Rheumatoid arthritis	1954	Female	No															5		Yes
Psoriatic arthritis	1975	Male	No															1 = No Pain	Hassle, side effects, addiction	Yes
Rheumatoid arthritis	1969	Female	No															8		Yes
Psoriatic arthritis	1945	Female	Yes	Topical (skin)	Years	No	Someone I know		Rarely	4	3	3	1: a little better	Pain	None	No		4	Hassle, cost	Yes
Rheumatoid arthritis	1994	Female	No															7	Safety	Yes
Psoriatic arthritis	1975	Male	No															3		Yes
Rheumatoid arthritis	1985	Female	Yes	Liquid, inhaled	Weeks	Yes	Dispensary		Twice a day	4	4	4	1: a little better	Stiffness, swelling	None	No		4	Safety	Yes
Rheumatoid arthritis	1987	Female	No															5		
Rheumatoid arthritis	2002	Female	No															3	Hassle	No
Rheumatoid arthritis	1984	Female	Yes	Topical (skin), capsule, liquid, oil, inhaled	Weeks	Yes	Dispensary	0-200 mg/day	Once a day	5	5	3	1: a little better	Pain	None	No		5		Yes
Rheumatoid arthritis	1954	Female	Yes	Inhaled	Years	Yes	Someone I know		Once a day	7	6	6	1: a little better	Stiffness, swelling, pain, fatigue	None	No		6	Cost	Yes
Psoriatic arthritis	1954	Male	Yes	Liquid	Weeks	Yes	Local store	200-500 mg/day	Twice a day	4	3	3	-1: a little worse	Swelling	None	No		5	Side effects	Yes
Rheumatoid arthritis	1970	Female	No															3	Hassle, addiction	Yes
Psoriatic arthritis	1971	Female	No															2	Safety, addiction	Yes
Rheumatoid arthritis	1976	Female	No															4	Cost	Yes
Rheumatoid arthritis	1992	Female	Yes	Inhaled	Years	Yes	Dispensary, Someone I know		Weekly	4	1 = No Pain	1 = no pain	2: much better	Stiffness, pain	None	No		2	Cost	Yes
Psoriatic arthritis	1945	Female	No															8	Side effects	Yes
Rheumatoid arthritis		Female	No															2	Cost, addiction	No
Psoriatic arthritis	1980	Female	No															3	Safety, hassle, side effects, addiction	Yes
Rheumatoid arthritis	1970	Female	No															2	Cost, side effects	Yes
Psoriatic arthritis	1973	Female	No															2		Yes
Rheumatoid arthritis	1955	Male	No															6	Cost	Yes
Rheumatoid arthritis	1949	Female	No															1 = No Pain		
Rheumatoid arthritis	1931	Female	No															2	Cost	No
Rheumatoid arthritis	1955	Female	No															8	Hassle, cost	Yes
Rheumatoid arthritis	1954	Male	No															2	Hassle	Yes
Rheumatoid arthritis	1934	Female																		
Psoriatic arthritis	1944	Male	No															3		Yes
Rheumatoid arthritis	1983	Female	No															2	Side effects	Yes
Rheumatoid arthritis	1979	Female	Yes	Oil	Months	Yes	Online	200-500 mg/day	Once a day	3	3	3	1: a little better	Stiffness, pain	None	No		3	Cost	Yes
Rheumatoid arthritis	1977	Female	No															4		Yes
Rheumatoid arthritis	1973	Female	No															1 = No Pain	Safety, hassle, cost	Yes
Rheumatoid arthritis	1964	Male	No															5		Yes
Rheumatoid arthritis	1943	Male	Yes	Topical (skin)	Years	Yes	Dispensary		Weekly	8	4	6	1: a little better	Pain	None	No		4	Cost	Yes
Psoriatic arthritis	1963	Female	Yes	Topical (skin), oil, inhaled	Months	No	Dispensary			5	3	4	1: a little better	Pain	None	No		5	Safety, hassle, cost, side effects	Yes
Rheumatoid arthritis	1961	Female	No															2	Safety, side effects	Yes
Rheumatoid arthritis	1955	Female	Yes	Inhaled	Months	Yes	Someone I know		Once a day	6	1 = No Pain	1 = no pain	2: much better	Pain	Pain	No		1 = No Pain		Yes
Rheumatoid arthritis	1950	Male	No															1 = No Pain		Yes
Psoriatic arthritis	1977	Male	No															3	Safety, hassle, cost, side effects, addiction	Yes
Psoriatic arthritis	1966	Male	No															2	Cost	Yes
Rheumatoid arthritis	1952	Female	No															2	Cost	Yes
Rheumatoid arthritis	1972	Female	No															2	Safety	Yes
Rheumatoid arthritis	1953	Female	No															2	Side effects	Yes
Rheumatoid arthritis	1949	Female	No															2		Yes
Psoriatic arthritis	1968	Male	No															2	Hassle	Yes
Rheumatoid arthritis	1977	Female	Yes	Capsule, liquid, oil, inhaled	Years	Yes	Dispensary, Someone I know	Over 1000 mg/day	Most days	9	5	5	2: much better	Stiffness, pain, fatigue	None	Yes	No	4	Hassle, cost	Yes
Rheumatoid arthritis	1991	Female	No															8	Safety, hassle, side effects, addiction	No
Rheumatoid arthritis	1978	Female	Yes	Oil	Years	Yes	Medical provider		Twice a day	8	1 = No pain	3	2: much better	Pain	None	Yes	No	6	Hassle, cost	Yes
Rheumatoid arthritis	1950	Female	No															3		Yes
Rheumatoid arthritis	1946	Female	No															6		Yes
Rheumatoid arthritis	1983	Male	No															2		No
Psoriatic arthritis	1967	Male	No															2	Safety, cost, side effects, addiction	Yes
Rheumatoid arthritis	1983	Female	No															2	Hassle, cost	Yes
Rheumatoid arthritis	1988	Female	No															5	Cost	Yes
Rheumatoid arthritis	1958	Female	No															5	Safety, side effects	Yes
Rheumatoid arthritis	1969	Female	No															2		Yes
Rheumatoid arthritis	1961	Female	No															2	Safety, side effects, addiction	Yes
Rheumatoid arthritis	1965	Female	No															3		Yes
Rheumatoid arthritis	1965	Female	No															6	Hassle	
Rheumatoid arthritis	1933	Female	No															4		Yes
Rheumatoid arthritis	1968	Female	No															2	Safety, cost, addiction	No
Rheumatoid arthritis		Female	No															2	Safety, hassle	Yes
Rheumatoid arthritis	1968	Female	No															7	Hassle, cost	Yes
Rheumatoid arthritis	1975	Female	No															5	Cost	Yes
Psoriatic arthritis	1943	Female	No															4	Side effects	Yes
Rheumatoid arthritis	1974	Female	No															3	Hassle	Yes
Rheumatoid arthritis	1966	Female	No															6	Side effects	No
